# A Novel Prime and Boost Regimen of HIV Virus-Like Particles with TLR4 Adjuvant MPLA Induces Th1 Oriented Immune Responses against HIV

**DOI:** 10.1371/journal.pone.0136862

**Published:** 2015-08-27

**Authors:** Ethan Poteet, Phoebe Lewis, Feng Li, Sheng Zhang, Jianhua Gu, Changyi Chen, Sam On Ho, Thai Do, SuMing Chiang, Gary Fujii, Qizhi Yao

**Affiliations:** 1 Michael E. DeBakey Department of Surgery, Division of Surgical Research, Baylor College of Medicine, Houston, TX, 77030, United States of America; 2 Houston Methodist Research Institute, Houston, TX, 77030, United States of America; 3 Molecular Express, Inc., Rancho Domínguez, CA, 90220, United States of America; 4 Center for Translational Research on Inflammatory Diseases (CTRID), Michael E. DeBakey VA Medical Center, Houston, TX, 77030, United States of America; University of Massachusetts Medical Center, UNITED STATES

## Abstract

HIV virus-like particles (VLPs) present the HIV envelope protein in its native conformation, providing an ideal vaccine antigen. To enhance the immunogenicity of the VLP vaccine, we sought to improve upon two components; the route of administration and the additional adjuvant. Using HIV VLPs, we evaluated sub-cheek as a novel route of vaccine administration when combined with other conventional routes of immunization. Of five combinations of distinct prime and boost sequences, which included sub-cheek, intranasal, and intradermal routes of administration, intranasal prime and sub-cheek boost (IN+SC) resulted in the highest HIV-specific IgG titers among the groups tested. Using the IN+SC regimen we tested the adjuvant VesiVax Conjugatable Adjuvant Lipid Vesicles (CALV) + monophosphoryl lipid A (MPLA) at MPLA concentrations of 0, 7.5, 12.5, and 25 μg/dose in combination with our VLPs. Mice that received 12.5 or 25 μg/dose MPLA had the highest concentrations of Env-specific IgG2c (20.7 and 18.4 μg/ml respectively), which represents a Th1 type of immune response in C57BL/6 mice. This was in sharp contrast to mice which received 0 or 7.5 μg MPLA adjuvant (6.05 and 5.68 μg/ml of IgG2c respectively). In contrast to IgG2c, MPLA had minor effects on Env-specific IgG1; therefore, 12.5 and 25 μg/dose of MPLA induced the optimal IgG1/IgG2c ratio of 1.3. Additionally, the percentage of germinal center B cells increased significantly from 15.4% in the control group to 31.9% in the CALV + 25 μg MPLA group. These mice also had significantly more IL-2 and less IL-4 Env-specific CD8^+^ T cells than controls, correlating with an increased percentage of Env-specific central memory CD4^+^ and CD8^+^ T cells. Our study shows the strong potential of IN+SC as an efficacious route of administration and the effectiveness of VLPs combined with MPLA adjuvant to induce Env specific Th1-oriented HIV-specific immune responses.

## Introduction

HIV envelope protein gp160, which is subsequently cleaved into gp120 (Env) and gp41, has been the focus of most vaccine candidates due to its location on the virus surface and essential role in binding the CD4 receptor [1]. The difficulty in targeting Env is that it has high sequence variability, post-processing variability, and mutates frequently [2,3]. With these characteristics in mind, the goal of an HIV vaccine is engineering a robust cytotoxic T Cell (CTL) response coupled with B cell generation of broadly neutralizing antibodies directed toward the CD4 binding site, thus attacking infected cells and preventing infection of additional cells [4–6].

Virus-like particles (VLPs) are replication-incompetent subunit vaccines that represent an intact, non-replicative virion lacking a genome, but maintaining the original antigenic composition of the Env proteins incorporated into the virion’s outer membrane. HIV VLPs have previously been shown to be potent immunogens which can directly activate B cells via the B cell receptor, or through the traditional pathway of presentation to dendritic cells or macrophages [7–10]. Previously, we have shown Simian Immunodeficiency Virus Gag plus HIV Env (SHIV) VLPs to be potent stimulators of humoral and systemic immune responses capable of generating robust CTL and humoral immune responses against SIV and HIV [11–13].

Although VLPs are capable of inducing an immune response without additional adjuvant, previous results have indicated that a robust response requires the addition of an adjuvant to the VLPs upon administration [14]. As subunit vaccines have increased in frequency, research into novel adjuvants has been carried out in parallel. Over the last two decades, adjuvants targeting the innate immune system, in particular the toll-like receptors (TLRs), have been developed to both activate the innate immune system and influence the adaptive immune response [15]. In particular, TLR4, which is expressed on antigen presenting cells, and the cytokine signaling of its proximal adaptor proteins, MyD88 and TRIF, are well studied [16]. In this study, we have used liposomes containing the TLR4 agonist monophosphoryl lipid A (MPLA), a predominantly TRIF-associated ligand, to amplify the immune response induced by our VLPs [17,18].

The route of administration affects the intensity, immunoglobulin class, and compartmentalization of the immune response, in particular as it is associated with the mucosal tissues [19,20]. Homologous intranasal administration has previously been shown to induce a global mucosal immune response as well as strong IgG and IgA titers in the mucosae [21,22]. Similarly, intradermal vaccination has been shown to produce both vaginal and rectal mucosal IgA and systemic IgG [23]. On the other hand, systemic immunization fails to generate mucosal IgA, but is still capable of inducing robust IgG titers [24,25]. IgG includes 4 sub-types broadly grouped into two categories, T helper 1 (Th1) and T helper 2 (Th2). One of these 4 sub-types is further divided between two homologues, IgG2a and IgG2c, which are 84% homologous; and in the case of C57BL/6 mice, only IgG2c is present [26]. A Th1 response favors IgG2a/IgG2c and IgG3, while a Th2 response favors IgG1, in mice [27]. However, Th1 and Th2 responses are more complex than merely humoral immune responses and are now more often associated with particular cytokines: Th1 includes cytokines IL-2, IL-12, and IFN-γ, and Th2 is associated with IL-4, IL-6, IL-10, and IL-13, among others [28,29]. Since their initial association with HIV, the Th1 and Th2 responses have become more loosely based on IgG subclasses and more focused on the cytokine profile, in favor of the Th1-associated cytokines [28,30]. Therefore, many vaccines have mixed Th1 or Th2 responses depending on their humoral- and cytokine-based immunity [31,32].

A cytokine profile induced by a vaccine is necessary to induce memory T cells and class-switched B cells. Central memory T cells are those T cells that have encountered an antigen, expanded into effector T cells, and finally consolidated into a small population of long lived central memory T cells [33]. Induction of central memory CD8^+^ T cells by vaccination is necessary to generate a long-term CTL response against the pathogen. Germinal centers are the sites of B cell maturation, where B cells undergo antibody hypermutation and class-switching, both necessary steps in the formation of broadly neutralizing antibodies [5,34]. The induction of both central memory T cells and germinal centers is necessary to generate the CTLs and the broadly neutralizing antibodies sought after for an effective HIV vaccine.

Our aim for this study was to analyze both the route of administration and adjuvant dose, and how these two variables influence IgA and IgG subtype class switching and global mucosal immunity. Considering the secondary effects of the humoral immune response, we measured the generation of memory CD8^+^ T cells and the formation of germinal center B cells. To do this, we used VLPs produced in a stable mammalian cell line with a fixed adjuvant (MPLA) concentration and immunized with various combinations of intradermal, intranasal, and sub-cheek inoculations. Based on these data, we chose the optimal route and then varied the MPLA concentration to optimize both route of administration and adjuvant for the production of germinal center B cells, memory T cells, and HIV specific antibodies.

## Materials and Methods

### Animals, antibodies, and reagents

Female C57BL/6 mice from Baylor College of Medicine were purchased and used at 8 weeks of age. All mice were maintained under specific pathogen-free conditions in the animal facilities of Baylor College of Medicine and in accordance with the animal protocol approved by Institutional Animal Care and Use Committee (IACUC). Flow cytometry antibodies were purchased from eBiosciences, BD Biosciences, and BioLegend. ELISA antibodies were purchased from Southern Biotech and Bethy Laboratories. Protein and peptide pools of HIV-1 Gag and Env were obtained from NIH AIDS Research and Reagents Program. Specific antibodies and proteins used, and peptide pool information are detailed in each of the following assay descriptions. The immunization adjuvant, VesiVax Conjugatable Adjuvant Lipid Vesicles (CALV) Kits, was obtained from Molecular Express, Inc. VesiVax CALV with no TLR4 Kit was used as control and VesiVax CALVs with TLR4 Kit at the indicated MPLA concentrations was used as primary adjuvant.

### Mammalian VLP production

Production of HIV VLPs followed a modified protocol based on studies described by Hammonds et al. [35]. Briefly, HIV-1 Gag/Env VLPs were produced from XC-18-derived cell lines engineered to express HIV-1 *gag* (HIV_IIIB_ strain) and *env* genes (HIV_BaL_ strain) under a tetracycline-inducible expression system (the cell lines are generous gifts from Dr. Spearman at Emory University). Cells engineered to produce HIV-1 Gag/Env VLPs were designated T-Rex Gag/Env. Cells were maintained in DMEM medium containing 10% Tet system-approved FBS, 4 mM L-glutamine, 100 units/ml penicillin, 100 μg/ml streptomycin, 100 μg/ml zeocin, and 5 μg/ml blasticidin. Production of VLPs was induced by adding 2 μg/ml of doxycycline once cells reached 90% confluency. Six days after induction, media containing VLPs were collected (25 ml/T-150 flask) and centrifuged twice at 2,000 x g for 5 min to remove cell debris. We then filtered the media through a 0.45 μm filter, and subjected it to ultracentrifugation at 140,000 x g for 2 h. The supernatant was carefully removed, and the remaining pellet, containing the VLPs, was resuspended in PBS (with Ca2^+^ and Mg2^+^), and stored at 4°C.

### Western blot

Western blot was performed as described previously [36]. Briefly, VLPs and recombinant proteins were solubilized in RIPA Buffer (Sigma, St. Louis, MO) and then in 2X Laemmli Buffer (Bio-Rad, Hercules, CA). After boiling the samples for5 minutes, we loaded them into a 10% SDS-PAGE gel and proceeded with electrophoresis for 2 hours at 100 volts. The protein was transferred to nitrocellulose for 2 hours at 90 volts, 4°C. Ponceau S stain (Sigma, St. Louis, MO) was used to verify protein transfer and the membrane was incubated overnight at 4°C with primary antibody, human monoclonal antibody to V3 of HIV-1 Env (447-52D; NIH AIDS Reagent Program). The following day, the membrane was washed 3 times in TBST (Tris-buffered saline plus Tween 20) and incubated for 2 hours at room temperature with anti-human HRP-conjugated secondary antibody (Southern Biotech, Birmingham AL). The secondary antibody was removed and the membrane washed 5 times with TBST, incubated with chemiluminescent substrate (GE, Schenectady, NY), and exposed to X-ray film (Denville Scientific, Metuchen, NJ). The film was developed with a Kodak X-GMAT 2000 (Eastman Kodak, Rochester, NY).

### Immunization

Two immunization regimens were used. The first was used to test multiple routes of immunization and consisted of one prime and two boosts via the routes specified. VLPs at a concentration of 4 mg/ml were mixed 1:1 v/v with VesiVax CALV having 300 μg/ml MPLA for a final concentration of 150 μg/ml of MPLA and 2 mg/ml of VLPs, and incubated at RT for 1 hour. The VesiVax CALV was supplied pre-conjugated to MPLA with cross-linking maleimide groups in neutral pH (7.0). The maleimide groups react with the sulfhydryl groups on the surface of the VLPs forming thioether bonds. Each mouse received 100 μg of VLPs and 7.5 μg of MPLA in a final volume of 50 μl, by the specified route. For all routes, mice were anesthetized before vaccine administration. For the intranasal (IN) route, 10 μl of VLPs were slowly applied to the anterior nares of the nasal cavity. The process was repeated 5 times for a total 50 μl of vaccine. For the intradermal (ID) route, the injection site was cleaned with ethanol before inoculation and each mouse received 50 μl of VLPs or PBS in the right hind quarter with a 1/2cc BD Ultra-Fine II insulin syringe (BD Biosciences, San José, CA). For the sub-cheek route (SC), 25 μl of VLPs or PBS were injected into each cheek (for a total volume of 50 μl) with a 1/2cc BD Ultra-Fine II insulin syringe (BD Biosciences, San Jose, CA). SC injection is a novel oral buccal cheek subcutaneous immunization route. We hypothesized that the high concentration of immune cells in the buccal mucosa and the abundance of lymph nodes around the neck region contribute to greater immune presentation of our intended antigen. The details of SC injection are illustrated in S1 Fig. Each mouse received one prime and two boosts via the indicated routes.

For the second immunization regimen, VLPs, at a concentration of 8 mg/ml, were mixed at a 1:1 v/v ratio with the indicated VesiVax CALV having 0, 300, 500, or 1000 μg/ml of MPLA. For each dose, this corresponded to (CALV(MPLA)): 0 (CALV), 7.5 (CALV(7.5)), 12.5 (CALV(12.5)), and 25 (CALV(25)) μg/dose, respectively. For the VLP only group, in which VLPs were not conjugated to VesiVax CALV, 8 mg/ml of VLPs was diluted 1:1 v/v in PBS (with Ca^2+^ and Mg^2+^). MPLA-only group received CALV(25). All samples were incubated for 1 hour at RT before inoculation. After anesthetizing the mice, we slowly applied 10 μl of VLPs to the anterior nares of the nasal cavity. The process was repeated 5 times for a total of 200 μg of VLPs in 50 μl of solution. Intranasal prime was administered on day 0, after which, one boost was delivered SC on each of the following days: 14, 28, and 42. For SC administration, mice were first anesthetized and then injected with 25 μl of VLPs or PBS into each cheek, for a total volume of 50 μl, with a 1/2cc BD Ultra-Fine II insulin syringe (BD Biosciences, San José, CA).

### Tissue collection

Mice were sacrificed on day 56 (week 8) by cervical dislocation and samples, including sera, vaginal wash, and splenocytes, were harvested. Approximately 500 μl of blood was drawn by heart puncture. Approximately 100 μl of blood was set aside for 1 hour to let it coagulate, and then centrifuged at 1000 x g, to separate out sera. Vaginal washes were collected by washing the vaginal tract several times with 100 μl of PBS (with Ca2^+^ and Mg2^+^) containing protease inhibitor. Spleens were extracted by sterile dissection. Single celled lymphocyte suspensions were prepared by mincing the spleens and then passing the tissue fragments through a 70 μm nylon mesh. Red blood cells (RBCs) from spleens were lysed with ammonium chloride potassium (ACK) RBC lysis solution.

### ELISA

Specific antibody levels in sera and vaginal washes were determined with an enzyme-linked immunosorbent assay (ELISA) kit, according to the manufacturer’s protocol (BD Biosciences, San José, CA). To detect Env- and Gag-specific antibodies, microtiter plates were coated with 2 μg/ml of VLPs, recombinant HIV_BaL_ gp120 (Env) protein, or recombinant HIV_IIIB_ Pr55 Gag (Gag) protein (NIH AIDS Research and Reference Reagent Program). Protein-coated plates were incubated overnight at 4°C. After discarding the coating solution, plates were washed 3 times with PBS + 0.05% Tween 20 (PBST), and blocked with PBS containing 5% bovine serum albumin (BSA), at RT for 2 hours. Sera or vaginal washes were diluted 1:100 or 1:20, respectively, with PBS, and 50 μl was added to each well. Plates were incubated overnight, washed 3 times with PBST, and incubated with goat anti-mouse IgA (Bethyl Laboratories, Montgomery TX), IgG (Southern Biotech, Birmingham AL), IgG1 (Southern Biotech, Birmingham AL), or IgG2c (Southern Biotech, Birmingham AL) horseradish peroxidase (HRP)-conjugated antibodies for 2 hours at RT. After washing any unbound conjugates out with PBST, TMB colorimetric substrate solution (Pierce, Rockford, IL) was added into each well. The HRP enzyme reaction was stopped with 100 μl of 2 N H_2_SO_4,_ and the OD values were read at 450 nm wavelength (against reference at 570 nm) in a microtiter reader (EL800, Bio-Tek Instruments, Winooski, VT).

### Intracellular cytokine staining

Splenocytes were seeded into 96-well plates at a density of 2 x 10^6^ cells/well. Brefeldin A (Sigma, St. Louis, MO) and monensin (Sigma, St. Louis, MO) added to each well at final concentrations of 10 μg/ml. Env or Gag peptide pool was added to each well at a concentration of 2 μg/ml. Phorbol 12-myristate 13-acetate (PMA; Sigma, St. Louis, MO) and ionomcyin (Sigma, St. Louis, MO) at concentrations of 50 ng/ml and 500 ng/ml, respectively, were the positive controls. Cells were incubated for 6 hours at 37°C, 5% CO_2_. Afterwards, cells were washed and incubated with anti-CD4 (clone: GK1.5; BD Biosciences, San José, CA) and anti-CD8a (clone: 53–6.7; BD Biosciences, San José, CA) cell membrane antibodies. After 45 minutes, cells were washed, fixed in BD Perm/Fix (BD Biosciences, San José, CA), permeabilized, and stained for intracellular cytokines IL-2 (clone: JES6-5H4; BD Biosciences, San José, CA), IL-4 (clone: 11B11; Ebiosciences, San Diego, CA), TNF-α (clone: MP6-XT22; Ebiosciences, San Diego, CA), and IFN-γ (clone: XMG1.2; BD Biosciences, San José, CA). Cells were incubated for an additional 60 minutes at RT, washed, and resuspended in BD Perm/Wash. The analysis of cytokines was done with an LSR-Fortessa (BD Biosciences San José, CA).

### Flow cytometry

Splenocytes and lymph node (LN) cells were resuspended in PBS containing 2% bovine serum albumin (BSA), 5 mM EDTA, and 0.03% NaN3 and added to 96-well conical-bottom plates, 1 x 10^6^ cells/well. The T cell panel included antibodies against mouse CD3e (clone: 145-2C11; BD Biosciences, San José, CA), CD4 (clone: GK1.5; BD Biosciences, San José, CA), CD8a (clone: 53–6.7; BD Biosciences, San José, CA), CD44 (clone: IM7; BD Biosciences, San José, CA), and CD62L (clone: MEL-14; BioLegend, San Diego, CA). The B cell panel included antibodies against mouse B220/CD45R (clone: RA3-6B2; BD Biosciences, San José, CA), CD3e (clone: 145-2C11; BD Biosciences, San José, CA), IgD (clone: 11–26; Ebiosciences, San Diego, CA), and GL-7/Ly-77 (clone: GL7; BD Biosciences, San José, CA). Cells were incubated with the primary antibody for approximately 1 hour at RT. Afterwards, they were fixed for 15 minutes in BD Perm/Fix, resuspended in BD Perm/Wash, and then analyzed with an LSR-Fortessa (BD Biosciences San José, CA). Central memory and effector memory T cells are defined as T cells (CD3^+^) expressing CD44^hi^ CD62L^+^ and CD44^hi^ CD62L^-^, respectively [37]. Germinal center B cells are defined as B cells (B220^+^ CD3^-^) with surface markers IgD^-^ and GL-7^+^, or CD95^+^ and GL-7^+^ [8,38].

### Atomic force microscopy (AFM)

To functionalize substrate samples with positive charges, a 10 μl droplet of (3-aminopropyl) triethoxysilane (APTES) (Sigma Aldrich) (0.1% v/v in DI water) was applied onto a freshly cleaved mica surface (Ted Pella Inc.). After 15 min at RT, the droplet was blown away. To prepare VLP samples on the functionalized substrate, 10 μl of VLP solution was applied onto the mica surface and incubated for 15 min at RT. The droplet was removed from the surface with nitrogen. The surface was rinsed with DI water, and dried again with nitrogen. The samples were analyzed with a multimode AFM M-8 (Bruker). All measurements were conducted in air at ambient conditions.

### Particle size analysis

The size distributions of the VesiVax CALV-VLP mixture, of VesiVax CALV only, and of VLP only were analyzed by measuring their dynamic light scattering with a Microtrac UPA 150 (Montgomeryville, PA). The samples were incubated for 1 hour at RT, and diluted for optimal reflected power with 100 mM sodium phosphate buffer (pH 7).

### Statistical analysis

Data from treated and control groups were analyzed and results presented as the arithmetic mean ± standard error mean (SEM). Pooled and individual samples were used for analysis as noted in the figure legends. By pooling the samples, the variance is decreased, weakening conclusions drawn from these experiments. Statistical analyses were done with Student’s unpaired t-test, 1-Way ANOVA, and Tukey post-hoc test, for comparison of multiple groups, or with 2-Way ANOVA and the Bonferonni post-hoc test for comparison of parametric data between two or more groups. The Kruskal-Wallis test or the Mann-Whitney test was used for non-parametric data. Graphpad Prism was used to calculate statistics (Graphpad Software, Inc., La Jolla, CA). A value of *p* < 0.05 was considered significant.

## Results

### Characterization of HIV VLPs and CALV conjugation

**Fig 1:**
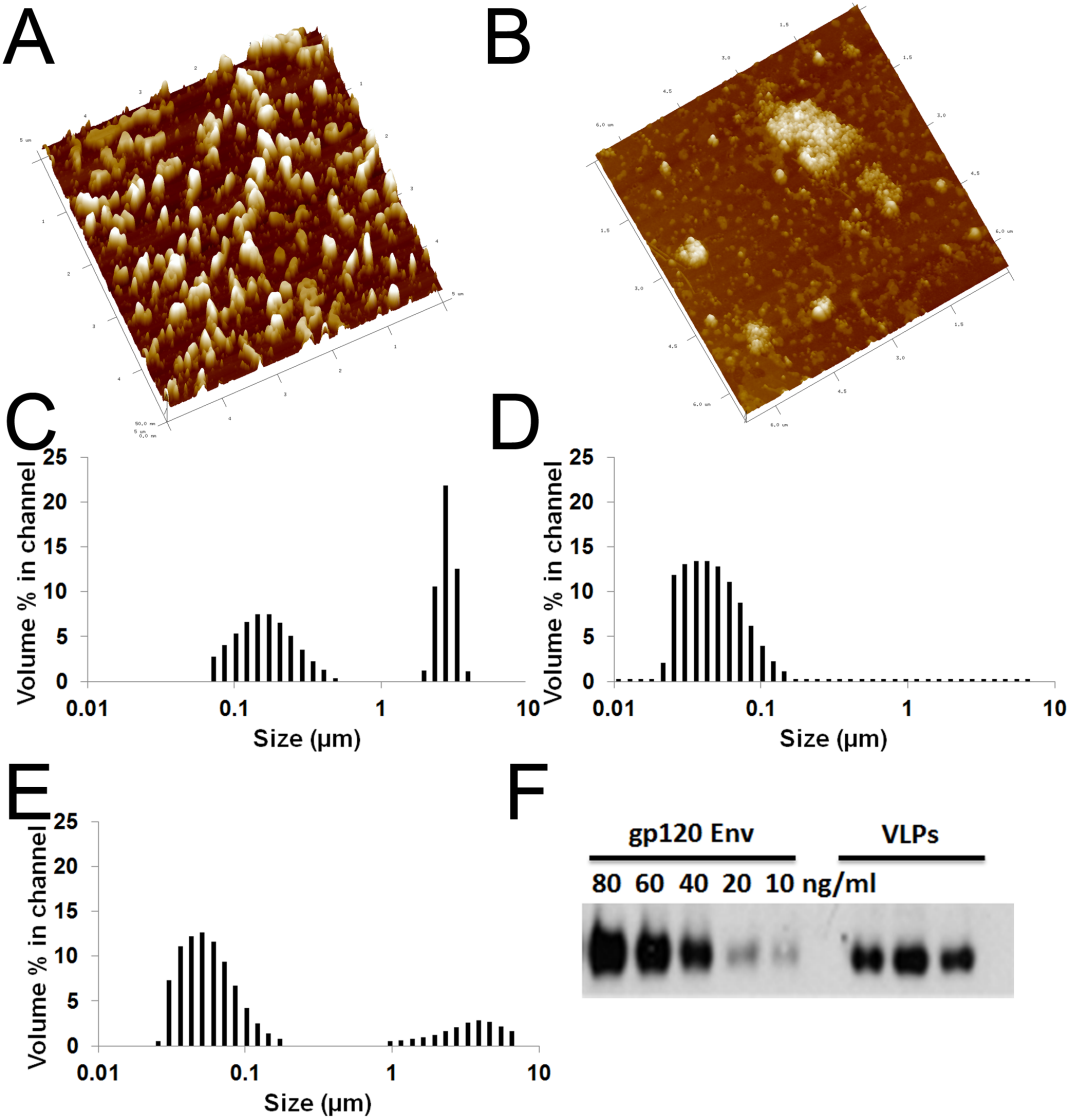
Characterization of VLPs and adjuvant. Atomic Force Microscopy of (A) VLPs only, measuring 40 nm in height and 171 nm in diameter, and (B) VesiVax CALV + VLPs, measuring 80 nm in height and 410 nm in diameter (scale: each dash is 200 nm). Size distribution by dynamic light scattering on a Microtrac UPA 150 of (C) VLPs only, (D) VesiVax CALV only, and (E) VesiVax CALV + VLPs. (F) Western blot of recombinant HIV_BAL_ gp120 at the indicated concentrations and 3 samples of VLPs (8 mg/ml) diluted 1:200 in RIPA buffer. Densitometric measurement of diluted VLPs yielded an average concentration of 39 ng/ml (39 ng/ml * 200 = 7.8 μg/ml undiluted).

VLPs were initially imaged with AFM to verify pseudovirus integrity and to differentiate differences in volume in the presence or absence of VesiVax CALV (Fig 1A and 1B). Although both singular VLPs and clustered VLPs were observed with AFM, measurements focused on a random sampling of singular VLPs with or without VesiVax CALV. On average, the sectional profiles of the singular VLPs measured 35 nm to 50 nm in height and 170 nm to 230 nm in diameter, while the VesiVax CALV + VLPs measured 45 nm to 90 nm in height and 200 nm to 350 nm in diameter. Dynamic light scatter was next used to estimate the percentage of singular versus clustered VLPs, and to confirm the average size of each. Similarly to the AFM results, the dynamic light scatter results showed that the two components of the VLP solution were singular VLPs of mean diameter of 145 nm (53% of volume) and clustered VLPs averaging 2.5 μm in diameter (47% of volume) (Fig 1C). When VLPs were combined with VesiVax CALV, no singular VLPs were observed; instead, clusters averaging 3.2 μm in diameter represented 20% of the total volume and the remaining 80% contained uncombined VesiVax CALV with a diameter of 48.6 nm (Fig 1D and 1E). To quantify VLP Env expression, Western blots of VLP lysate diluted 1:200 were run alongside a recombinant gp120 Env standard curve between 10 ng/ml and 80 ng/ml (Fig 1F). Densitometric measurement of diluted VLPs yielded an average concentration of 39 ng/ml, which corresponded to 7.8 μg/ml undiluted (39 ng/ml * 200). The average concentration of 7.8 μg/ml of Env resulted in 1.56 μg of Env in each VLP vaccine dose of 200 μg.

### Intranasal prime and sub-cheek boost generates a robust IgG response

**Fig 2:**
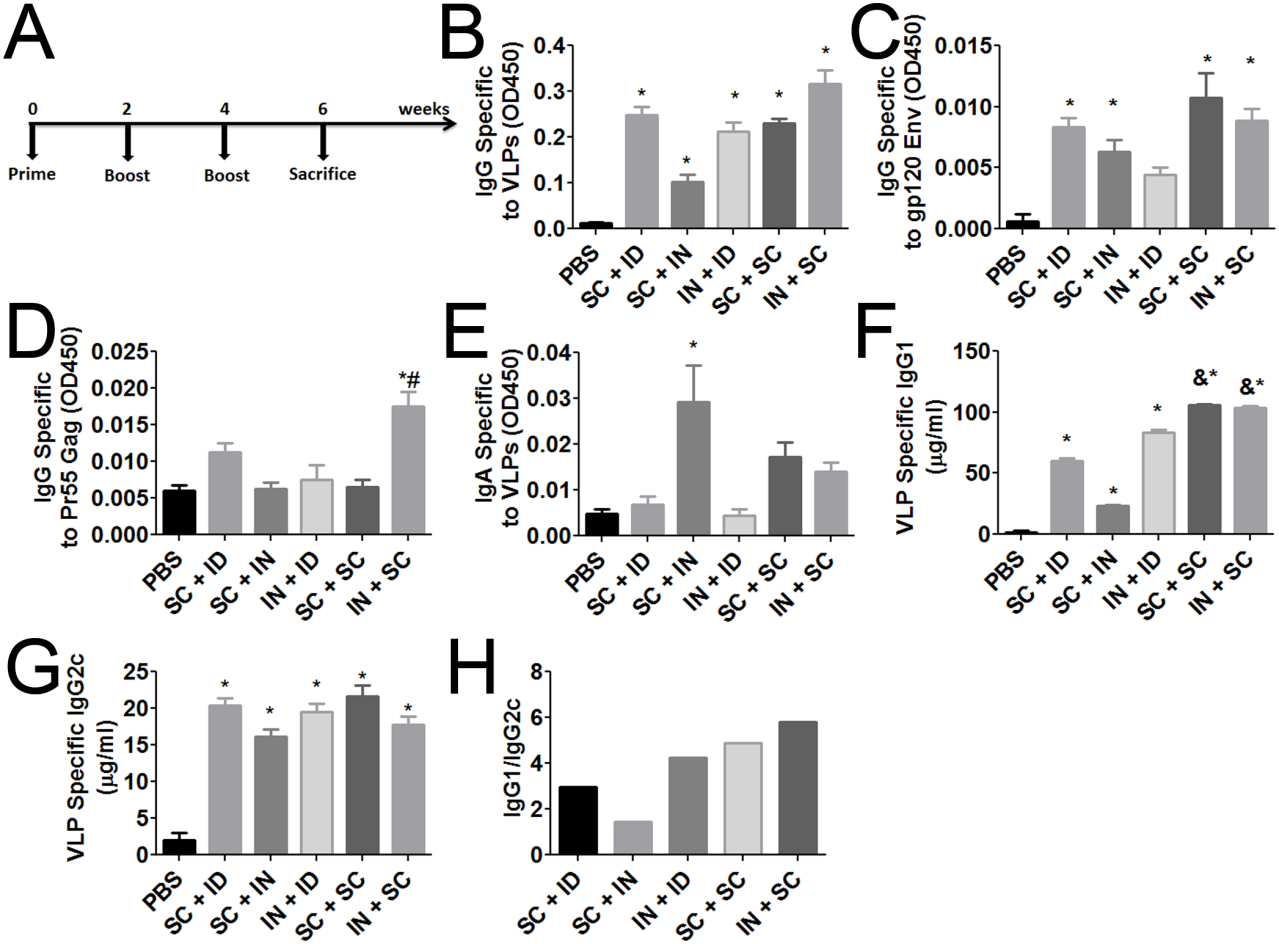
Specific sera IgG and mucosal IgA titers in mice treated with different prime-boost strategies. (A) Immunization regimen for evaluation of route of administration. ELISA plates were coated with 2 μg/ml of the indicated target protein. Sera from individual mice at time of sacrifice were diluted 1:100 and specific IgG titers determined against (B) VLPs, (C) gp120 Env, and (D) Pr55 Gag. (E) VLP-specific IgA in mucosal wash (1:20 dilution) from time of sacrifice. (F) IgG1 quantitative ELISA of pooled sera (duplicates repeated in triplicate) specific to VLPs (G) IgG2c quantitative ELISA of pooled sera (duplicates repeated in triplicate) specific to VLPs (H) Ratio of mean IgG1 to mean IgG2c of the indicated immunization groups. Error bars represent mean ± SEM (n = 5 for B, C, D, and E; n = 3 for F and G); * *p*<0.05 (1-way ANOVA and Tukey Post-Hoc tests versus PBS). # *p*<0.05 (1-way ANOVA and Tukey Post-Hoc tests versus all other groups). & *p*<0.05 (1-way ANOVA and Tukey Post-Hoc tests versus SC + ID, SC + IN, and IN + ID).

We compared five different routes of VLP immunization by coupling sub-cheek injections with the well-studied intradermal and intranasal routes of administration in the following combinations: sub-cheek prime plus intradermal boost (SC+ID), sub-cheek prime plus intranasal boost (SC+IN), intranasal prime plus intradermal boost (IN+ID), sub-cheek prime plus sub-cheek boost (SC+SC), and intranasal prime plus sub-cheek boost (IN+SC). To enhance the immune response, all VLPs were coupled to VesiVax CALV containing 7.5 μg/dose MPLA. Mice received one prime and then two boosts each spaced two weeks apart. Mice were sacrificed two weeks after their final boost (Fig 2A). All immunizations resulted in VLP-specific IgG titers that were higher than the PBS control titers [optical density (OD) of 0.01]. Immunization via IN+SC resulted in the highest titer (0.31 OD) (Fig 2B). All groups also showed HIV_BaL_ Env-specific antibody titers that were significantly higher than those of PBS control, except the IN+ID group (Fig 2C). Finally, IN+SC was the only immunization route that resulted in a significant specific antibody response (0.018 OD) to Pr55 Gag (PBS control: 0.006 OD) (Fig 2D). The vaginal mucosa VLP-specific IgA antibody response was measured at time of sacrifice. Only immunization via SC+IN resulted in a significant increase of IgA specific to VLPs (0.029 OD) (PBS control: 0.005 OD) (Fig 2E).

To determine IgG class switching, IgG1 and IgG2c quantitative ELISAs against VLPs were conducted with pooled sera in conjunction with a standard curve. The IgG1 specific to VLPs differed dramatically between the route of immunization groups, with SC+IN producing the lowest of 23.3 μg/ml and SC+SC and IN+SC reporting 106 μg/ml and 104 μg/ml respectively (Fig 2F). All immunized groups had significantly greater titers compared to PBS control (1.38 μg/ml). IgG2c titers were similar between groups with concentrations ranging from 16.1 μg/ml for SC+IN to 21.7 μg/ml for SC+SC (Fig 2G). The ratio of IgG1 to IgG2c was closest to 1.0 with SC+IN (1.45) and furthest from 1.0 in SC+SC (4.90) and IN+SC (5.82) due to both SC+SC and IN+SC having higher overall titers (Fig 2H). Based on the route of administration data, both SC+SC and IN+SC produced the best immune responses and additional experiments were conducted with IN+SC due to high titers, as well as it was the only group to produce significant antibodies against Gag.

### CALV-MPLA-HIV VLP stimulates antibody production

**Fig 3:**
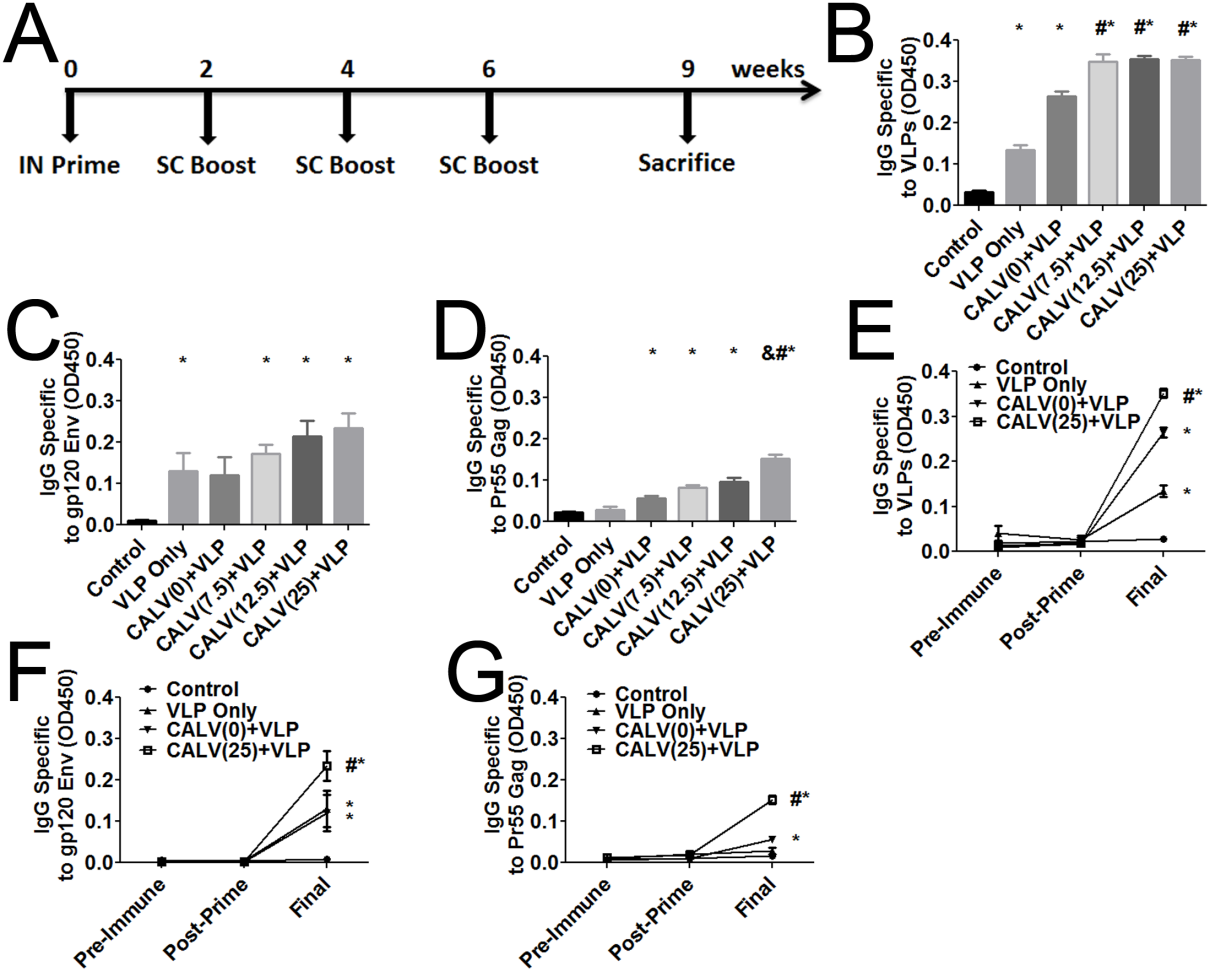
Immunization regimen and ELISA antibody titers to VLPs, HIV-1 Bal gp120 Env, and HIV-1 IIIB Pr55 Gag in mice immunized with VLPs. (A) Immunization regimen for MPLA adjuvant study. ELISA plates were coated with 2 μg/ml VLPs, gp120 Env, or Gag incubated with individual mouse sera diluted 1:100 and probed for IgG specific to (B) VLPs, (C) gp120 Env, and (D) Pr55 Gag. * *p*<0.05 (1-way ANOVA and Tukey Post-Hoc tests versus control group). # *p*<0.05 (1-way ANOVA and Tukey Post-Hoc tests versus VLP-only and CALV(0)+VLP groups). & *p*<0.05 (1-way ANOVA and Tukey Post-Hoc tests versus CALV(7.5)+VLP and CALV(12.5)+VLP groups). Time course (pre-immunization, post-prime, and at time of sacrifice (final)) of IgG antibodies from individual mice against (E) VLPs, (F) gp120 Env, and (G) Pr55 Gag. Error bars represent mean ± SEM (n = 8); * *p*<0.05 (2-way ANOVA and Bonferroni Post-Hoc tests versus control group). # *p*<0.05 (2-way ANOVA and Bonferroni Post-Hoc tests versus VLP-only and CALV(0)+VLP).

After determining the optimal route, we sought to investigate the immunogenicity of VesiVax CALV(MPLA)+VLP vaccine with varying MPLA adjuvant concentrations: 0 {CALV(0)+VLP}, 7.5 {CALV(7.5)+VLP}, 12.5 {CALV(12.5)+VLP}, and 25 {CALV(25)+VLP} μg/dose. An intranasal prime followed by three sub-cheek boosts was our immunization strategy (Fig 3A). Serum IgG was measured in each mouse before immunization, post-prime, and after the final boost. IgG titers, as determined by ELISA, showed a differential increase in VLP-specific IgG (Fig 3B) for all VLP immunogen groups. Titers after immunization with VLPs alone (0.135 OD), CALV(0)+VLP (0.265 OD), CALV(7.5)+VLP (0.348 OD), CALV(12.5)+VLP (0.354 OD), and CALV(25)+VLP (0.351 OD) were significantly higher than those of the control group (0.032 OD). Furthermore, the addition of MPLA to the liposome resulted in VLP-specific IgG titers that were significantly higher than that obtained with VLP alone or with VLP conjugated to an empty liposome without MPLA {CALV(0)+VLP}.

We next ascertained the specificity of the mouse sera to recombinant HIV-1 Pr55 Gag and HIV-1 gp120 Env, the two proteins that form the VLPs (Fig 3C and 3D). Env-specific titers were significantly higher than those of the control group (0.011 OD) in the VLP-only (0.130 OD), CALV(7.5)+VLP (0.173 OD), CALV(12.5)+VLP (0.214 OD), and CALV(25)+VLP (0.234 OD) groups, but there was no significant difference between CALV(0)+VLP (0.120 OD) and control. Gag-specific titers in the CALV(0)+VLP (0.057 OD), CALV(7.5)+VLP (0.083 OD), CALV(12.5)+VLP (0.096 OD), and CALV(25)+VLP (0.125 OD) groups were significantly higher than those of the control group (0.021 OD). In addition, the IgG level in the CALV(25)+VLP group was significantly greater than that of both the VLP-only and CALV(0)+VLP group, as well as those of the groups with lower MPLA concentrations, CALV(7.5)+VLP and CALV(12.5)+VLP.

Sera were collected before immunization, after prime, and at time of sacrifice to evaluate the effect of both the prime and the boost on IgG titers to VLP, Env, Gag, respectively (Fig 3E, 3F and 3G). No significant difference was detected in pre-immunization and post-prime levels of IgG between VLP-containing groups (VLP-only, CALV(0)+VLP, and CALV(25)+VLP) and control regarding antibodies specific to VLPs, Gag, or Env. However, there was a significant difference in the anti-VLP and the anti-Env IgG titers at the final time point between control (0.028 OD VLP; 0.008 OD Env) and VLP-only (0.135 OD VLP, 0.130 Env), CALV(0)+VLP (0.265 OD VLP; 0.120 Env), and CALV(25)+VLP groups (0.351 OD VLP; 0.234 Env). Only CALV(0)+VLP (0.057 OD) and CALV(25)+VLP (0.153 OD) immunizations resulted in a significantly increased response against Gag, relative to control response at the final time point (0.017 OD). Therefore, CALV (25)+VLP immunization resulted in VLP, Gag, and Env-specific titers that were significantly higher than those observed in the VLP-only and CALV(0)+VLP groups at the final time point.

### CALV(MPLA)+VLPs stimulate IgG2c class switching

**Fig 4:**
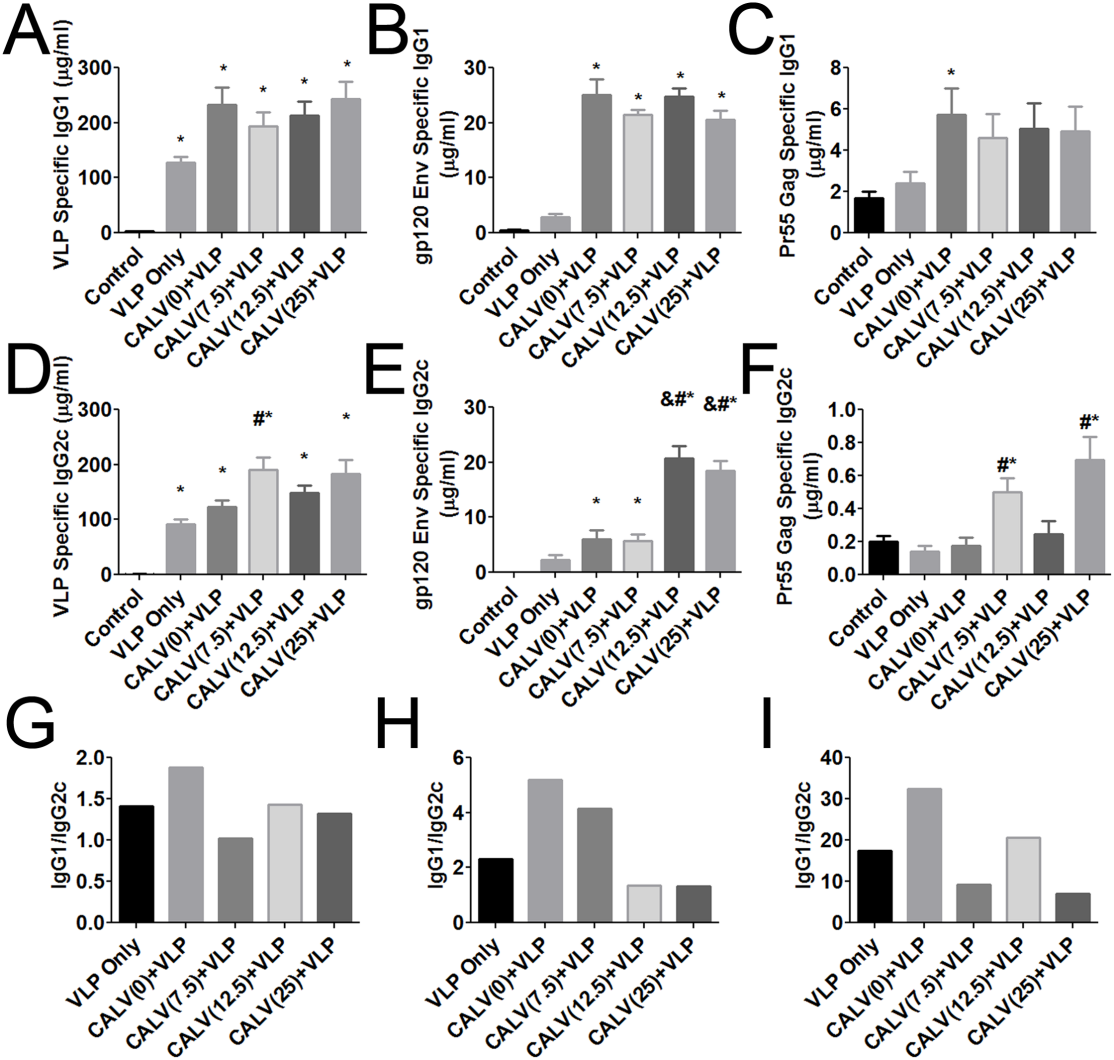
IgG1 and IgG2c sera titers against VLPs, HIV-1 Bal gp120 Env, and HIV-1 IIIB Pr55 Gag in mice immunized with VLPs. ELISA plates were coated with 2 μg/ml VLPs, gp120 Env, or Pr55 Gag. IgG1 quantitative ELISA of pooled mouse sera (duplicates repeated in triplicate) specific to (A) VLPs, (B) gp120 Env, and (C) Pr55 Gag. IgG2c quantitative ELISA of pooled mouse sera (duplicates repeated in triplicate) specific to (D) VLPs, (E) gp120 Env, and (F) Pr55 Gag. Ratio of mean concentration of IgG1 to mean concentration of IgG2c for (G) VLPs, (H) gp120 Env, and (I) Pr55 Gag. * *p*<0.05 (1-way ANOVA and Tukey Post-Hoc tests versus control group). Error bars represent mean ± SEM (n = 3); # *p*<0.05 (1-way ANOVA and Tukey Post-Hoc tests versus VLP-only and CALV(0)+VLP groups). & *p*<0.05 (1-way ANOVA and Tukey Post-Hoc tests versus CALV(7.5)+VLP).

We next determined the concentrations of IgG subclasses of antibodies specific to VLPs, Env, and Gag that had been induced by VesiVax CALV conjugated to MPLA and VLPs in mice. The results of IgG1 ELISA against VLPs of mouse sera from time of sacrifice showed a significant increase in IgG1 antibodies in all groups immunized with VLPs [VLP-only (128 μg), CALV(0)+VLP (233 μg), CALV(7.5)+VLP (194 μg), CALV(12.5)+VLP (213 μg), and CALV(25)+VLP (243 μg)] (control 2.8μg) (Fig 4A). This trend was repeated for IgG1 antibodies directed against Env except that VLPs alone did not induce a significant difference between controls (Fig 4B). Against Gag, only the IgG1 antibody titer of the CALV(0)+VLP (5.70 μg) was significantly higher than that of the control group (1.71 μg) (Fig 4C). Therefore, significant anti-VLP and anti-Env specific IgG1 antibodies were induced by CALV(MPLA)+VLPs IN+SC immunization strategy.

The IgG2c antibody titers against VLPs were similar to those of IgG1. The VLP-specific titers of VLP Only (91 μg), CALV(0)+VLP (124 μg), CALV(7.5)+VLP (191 μg), CALV(12.5)+VLP (149 μg), and CALV(25)+VLP (184 μg) groups were all still significantly higher than control response (0.310 μg) (Fig 4D). In addition, CALV(7.5)+VLP was significantly greater than both VLP only and CALV(0)+VLP. For Env specific antibodies, CALV(0)+VLP (6.05 μg), CALV(7.5)+VLP (5.68 μg), CALV(12.5)+VLP (20.7 μg), and CALV(25)+VLP (18.4 μg) groups induced responses that were significantly higher than control’s (0.024 μg) (Fig 4E). Also, Both CALV(12.5)+VLP and CALV(25)+VLP were significantly greater than VLP only, CALV(0)+VLP, and CALV(7.5)+VLP. Finally, against Gag, CALV(7.5)+VLP (0.501 μg) and CALV(25)+VLP (0.696 μg) showed a response that was significantly higher than the control (0.201 μg), and VLP only (0.139 μg) and CALV(0)+VLP (0.177 μg) groups (Fig 4F). IgG1 was predominant for all ratios of IgG1 to IgG2c for antibodies specific to VLPs, Env, and Gag (Fig 4G, 4H and 4I). Against Env, in contrast to CALV(0)+VLP (5.18 IgG1/IgG2c), both CALV(12.5)+VLP (1.35 IgG1/IgG2c) and CALV(25)+VLP (1.31 IgG1/IgG2c) had ratios approaching 1, which indicates CALV adjuvant has the potential to tilter VLP immunization towards Th1 immune response.

### IN+SC regimen induces a slight increase of the mucosal IgA antibody response

**Fig 5:**
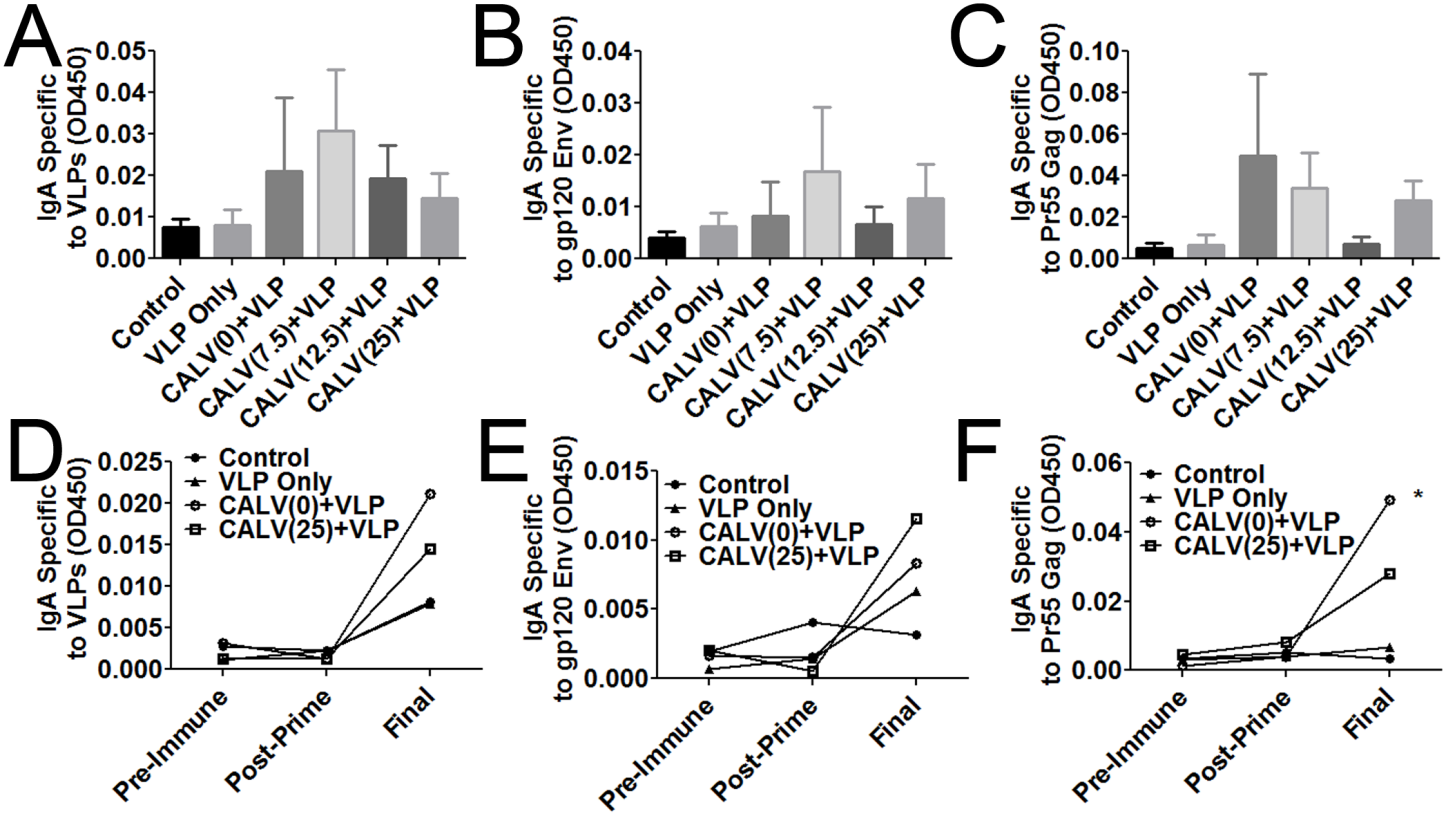
Mucosal IgA from vaginal wash. ELISA plates were coated with 2 μg/ml of the indicated target protein. Individual vaginal washes were diluted 1:20 in PBS and tested for IgA against (A) VLPs, (B) gp120 Env, and (C) Pr55 Gag. Washes collected before immunization, after intranasal prime, and at time of sacrifice (final) of IgA against (D) VLPs, (E) gp120 Env, and (F) Pr55 Gag. Error bars represent mean ± SEM (n = 8); * *p*<0.05 (2-way ANOVA and Bonferroni Post-Hoc tests versus control and CALV(0)+VLP groups).

We did not observe significant titers of mucosal IgA in our initial route of administration experiments that tested the IN+SC vaccine regimen (Fig 2E). Vaginal washes from each mouse were taken before immunization, after intranasal prime, and at time of sacrifice to measure mucosal antigen-specific IgA levels. When testing final mucosal IgA titers against VLPs, Env, and Gag in all immunization groups, we observed that, although no statistical significant difference was reached between CALV+VLP vaccinations and the control group, some strong responders did exist in each group (Fig 5A, 5B and 5C).

Temporally, there was no difference between pre-immune and post-prime titers, but the titers increased after the final boost in all groups immunized with VLPs. However, this increase didn’t reach statistical difference. On the other hand, the IgA level of Gag-specific antibodies in the CALV(0)+VLP group was significantly higher than those of the control and VLP-only groups, at the final time point (Fig 5D, 5E and 5F).

### CALV(MPLA)+VLP immunization enhances germinal center B cell induction

**Fig 6:**
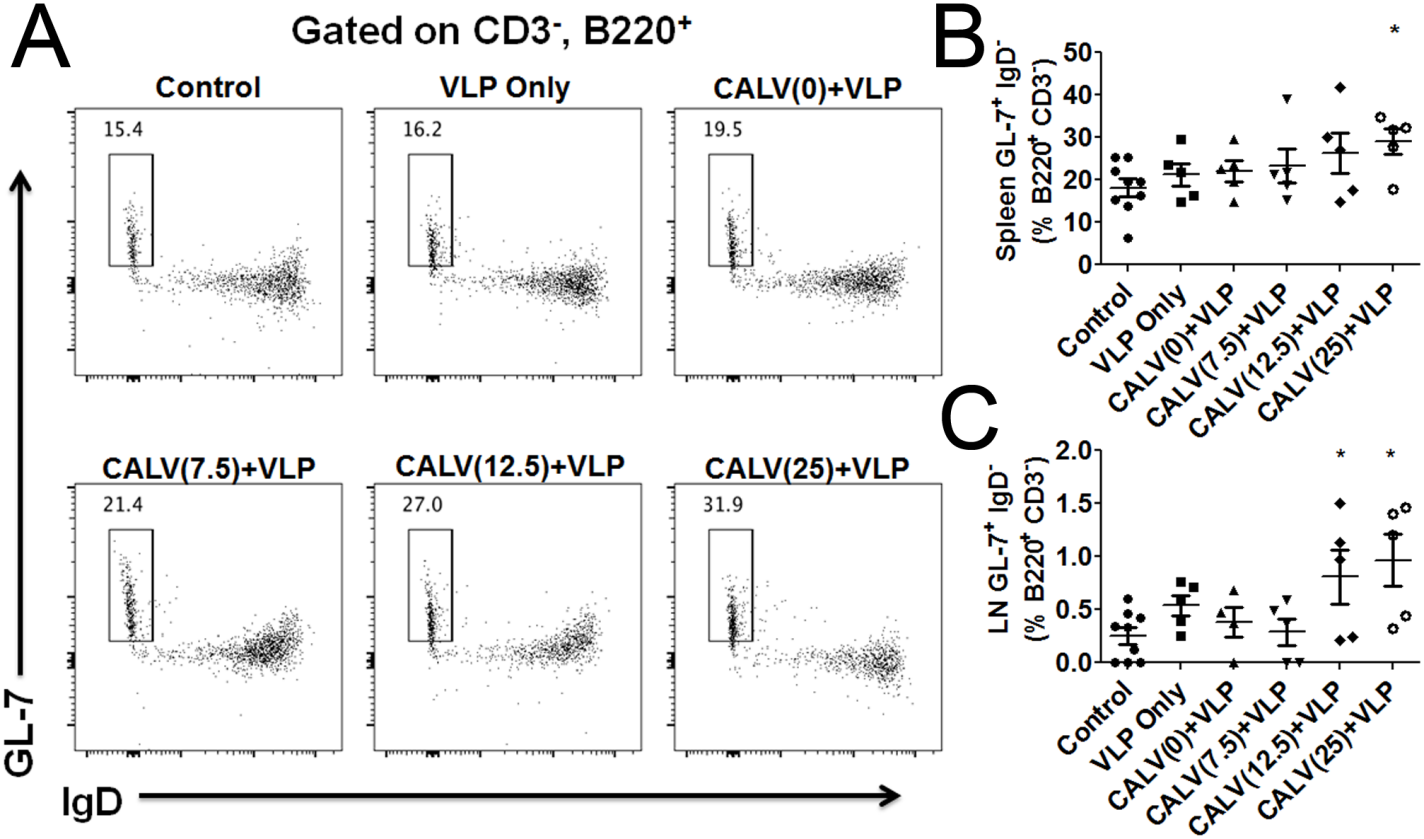
Germinal center B cells in the spleen and lymph nodes (LN). (A) Representative flow cytometry dot plots of the germinal center B cells formed in mice spleen immunized with the indicated vaccine at time of sacrifice. (B) Percentage of B220^+^CD3^-^ that are GL-7^+^ and IgD^-^ in the spleens of mice in each immunization group. (C) Percentage of B220^+^CD3^-^ that are GL-7^+^ and IgD^-^ in the lymph nodes of mice in each immunization group. Error bars represent mean ± SEM (n = 6); * p<0.05 (Student unpaired t-test versus control).

We determined the relative proportions of cell populations of germinal center B cells (B220^+^, CD3^-^, GL-7^+^, IgD^-^) isolated from spleen and lymph nodes. Shown in a representative splenocytes analysis (Fig 6A), the percentage of germinal center B cells in the CALV(25)+VLP group is 31.9% and the control group is 15.4%. Fig 6B showed a statistical analysis on percentage of splenocytes germinal center B cells among different immunization groups, the CALV(25)+VLP group (31.9%) was significantly higher than that of the control group (15.4%). Although both CALV(7.5)+VLP (23.3%) and CALV(12.5)+VLP (27.0%) groups had higher mean percentages of germinal center B cells, these trends did not reach statistical significance when compared with control values. However, the percentage of germinal center B cells from the lymph nodes of mice in the CALV(12.5)+VLP (0.81%) and CALV(25)+VLP (0.96%) groups were significantly higher than those of control mice (0.25%) (Fig 6C).

### CALV(MPLA)+VLP immunization enhances Env-specific CD8^+^ T cells with high IL-2 and reduced IL-4 production

**Fig 7:**
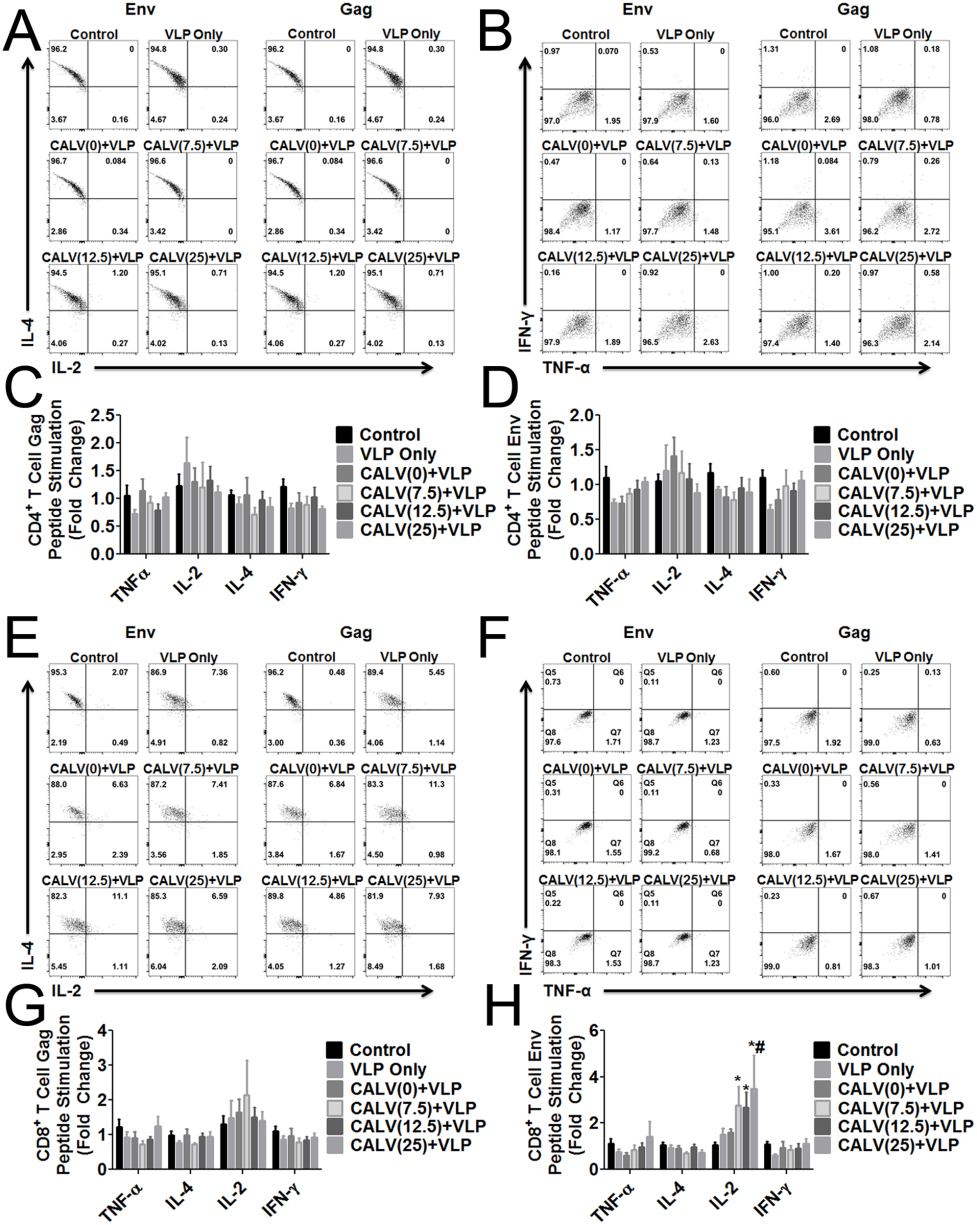
Intracellular cytokine staining of mouse splenocytes stimulated with HIV-1 Consensus B Env and Gag peptide pools. (A) Representative dot plot of CD4^+^ T cells expressing IL-2 and IL-4 after stimulation with HIV-1 Consensus B Env and Gag peptide pools. (B) Representative dot plot of CD4^+^ T cells expressing TNF-α and IFN-γ after stimulation with HIV-1 Consensus B Env and Gag peptide pools. (C) Relative levels of IL-2, IL-4, TNF-α, and IFN-γ in CD4^+^ T cells after stimulation with 2 μg/ml Gag peptide pool standardized to PBS control. (D) Relative levels of IL-2, IL-4, TNF-α, and IFN-γ in CD4^+^ T cells after stimulation with 2 μg/ml Env peptide pool standardized to PBS control. (E) Representative dot plot of CD8^+^ T cells expressing IL-2 and IL-4 after stimulation with HIV-1 Consensus B Env and Gag peptide pools. (F) Representative dot plot of CD8^+^ T cells expressing TNF-α and IFN-γ in CD8^+^ T cells after stimulation with HIV-1 Consensus B Env and Gag peptide pools. (G) Relative levels of IL-2, IL-4, TNF-α, and IFN-γ after stimulation with 2 μg/ml Gag peptide pool standardized to PBS control. (H) Relative levels of IL-2, IL-4, TNF-α, and IFN-γ in CD8^+^ T cells after stimulation with 2 μg/ml Env peptide pool standardized to Control. Error bars represent mean ± SEM (n = 6); * *p*<0.05 (2-Way ANOVA and Bonferroni Post-hoc tests versus control group). # *p*<0.05 (2-Way ANOVA and Bonferroni Post-hoc tests versus VLP Only and CALV(0)+VLP groups).

To characterize the cellular immune response specific to HIV Gag or Env, mice were sacrificed and splenocytes harvested and stimulated with 2 μg/ml of Env or Gag peptide pools. Induction of IL-2, IL-4, TNF-α, and IFN-γ, in both CD4^+^ and CD8^+^ T cells, was compared between the immunized groups. Incubation of CD4^+^ T cells with Env or Gag peptide pools resulted in no change in IL-2, IL-4, TNF-α, or IFN-γ expression (Fig 7A, 7B, 7C and 7D). On the other hand, incubation of CD8^+^ T cells with Env peptide pools resulted in a significant fold increase in IL-2, which depended on the immunization the mice had received: CALV (7.5)+VLP (2.7 fold), CALV(12.5)+VLP (2.7 fold), and CALV(25)+VLP (3.4 fold) (Fig 7E and 7G). Moreover, mice immunized with CALV(25)+VLP had significantly more IL-2^high^ Env-specific CD8^+^ T cells than mice immunized with CALV(0)+VLP. Furthermore, IL-2 response was multivariate; after CALV(25)+VLP immunization, mice showed increased numbers of CD8^+^ T cells that produced more IL-2 and less IL-4 than controls. IL-2 expression in response to Gag peptide pool was not significantly different between any of the groups (Fig 7H). No changes were observed in the expression of TNF-α or IFN-γ between any of the groups (Fig 7F, 7G and 7H).

### CALV(MPLA)+VLP immunization increases central memory T Cells

**Fig 8:**
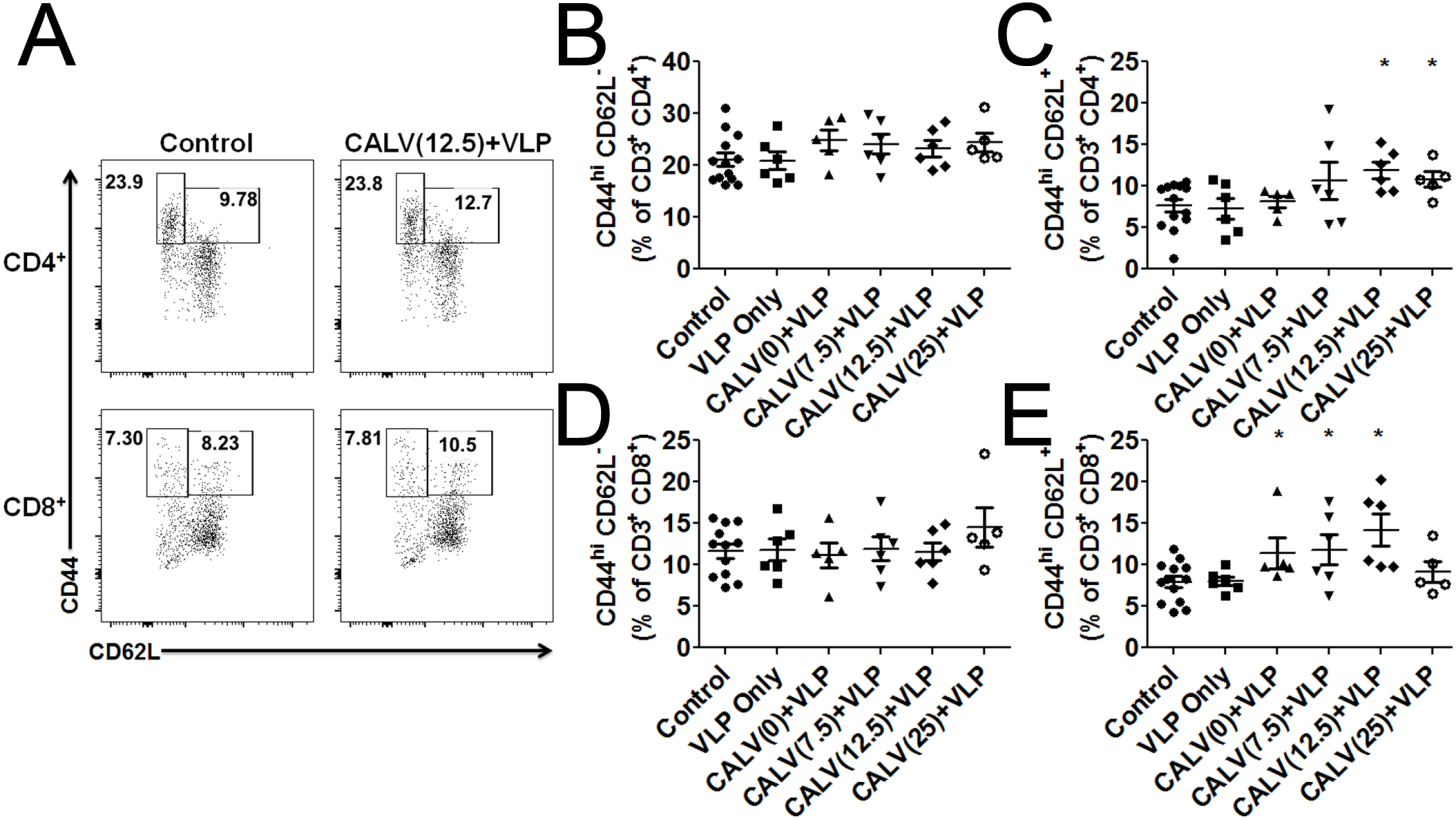
Effector (CD44^hi^, CD62L^-^) and central (CD44^hi^, CD62L^+^) memory T cell subsets in mouse CD3e^+^ and either CD4^+^ or CD8a^+^ splenocytes. (A) Representative dot plot of effector and central memory of CD4^+^ and CD8a^+^ T cells. (B) Percentage of CD4^+^ cells expressing high levels of CD44 and no detectable levels of CD62L in each immunization group. (C) Percentage of CD4^+^ cells expressing high levels of CD44 and CD62L. (D) Percentage of CD8a^+^ cells expressing high levels of CD44 and no detectable levels of CD62L in each immunization group. (E) Percentage of CD8a^+^ cells expressing high levels of CD44^+^ and CD62L^+^ staining. Error bars represent mean ± SEM (n = 6); * indicates p<0.05 (Student’s unpaired t-test when compared to control group).

Central memory and effector memory T cells are defined as CD44^hi^ CD62L^+^ and CD44^hi^ CD62L^-^, respectively. No change in CD4^+^ or CD8^+^ effector T cells was observed in any group: approximately 24% of CD4^+^ T cells were classified as effector memory T cells and 8% of CD8^+^ T cells as effector memory T cells (Fig 8B and 8D). However, the percentage of central memory CD4^+^ T cells trended upwards in all groups immunized with VLPs and liposomal conjugates, but this increase was significant only in CALV(12.5)+VLP (11.9%) and CALV(25)+VLP groups (10.2%) (control: 7.62% CD4^+^ T cells) (Fig 8A–8C). The percentage of central memory of CD4^+^ T cells in mice immunized with CALV(0)+VLP (8.1%) or CALV(7.5)+VLP (10.7%) also increased, but was not significantly different than that of control values. The percentage of central memory CD8^+^ T cells also increased significantly in mice immunized with CALV(0)+VLP (11.4%), CALV(7.5)+VLP (11.8%), and CALV(12.5)+VLP (14.2%) (Control: 7.92%) (Fig 8E). No differences were detected between the control group and VLP-only (8.33%) or CALV(25)+VLP groups (9.14%).

## Discussion

The aim of this study was to evaluate our novel route of immunization using mammalian system produced VLPs in conjunction with an MPLA conjugated liposomal adjuvant. We have previously demonstrated the effectiveness of baculovirus derived SHIV VLPs in inducing an HIV specific immune response in mice [39]. Our previous studies were accomplished with traditional routes of administration (e.g. intradermal and intraperitoneal) and only intermittently employed an adjuvant in addition to the VLP itself, which has been shown to have immunogenic properties [8,11]. Here, we have validated our mammalian VLPs with the novel route of sub-cheek immunization in combination with an MPLA liposomal adjuvant dose response to effectively induce a Th1-like immune response in mice.

Based on investigations in HIV-1 Highly Exposed Persistently Seronegative (HEPS) individuals, focus has centered on three major immune responses as criteria for evaluating the efficacy of an HIV vaccine: 1) A strong mucosal memory response to HIV, 2) An HIV-1 specific CD8^+^ T cell response, and 3) The induction of CD4 binding site (CD4bs)-specific broadly neutralizing antibodies [40,41]. Below, we address each of these criteria individually in the context of our HIV-1 VLPs novel route of administration and VesiVax CALVs containing MPLA. We have also taken into account new findings from human vaccine trials.

A strong mucosal response was believed to be a first-line of defense against HIV-1 transmitted through sexual intercourse. However, the most recent HIV-1 vaccine trial, RV144, reported that high serum IgA inversely correlated with vaccine efficacy [42,43]. Further analysis revealed that HIV-specific serum IgA interfered with antibody-dependent cell mediated cytotoxicity (ADCC), which is an IgG-mediated process [43,44]. This is further complicated by the fact that humans have two subtypes of IgA antibodies, IgA_1_ and IgA_2_, while mice do not have IgA subtypes [45]. Because mice lack the FcαR, they have lower IgA serum levels than humans do, and primarily have monomeric IgA. Additional studies are required to determine whether mouse IgA is capable of interfering with ADCC as human IgA does [46,47]. Therefore, our study focused on mucosal IgA, of which our vaccine induced minimal specific titers in all cases using sub-cheek administration either as a prime or as boost, or when the MPLA concentration was varied. Only the immunization regimen of intranasal prime plus intradermal boost resulted in specific mucosal IgA titers. Intranasal vaccination and MPLA have been shown to induce a strong IgA response; therefore, the most likely cause of low vaginal IgA titers is the sub-cheek administration [48]. Because our focus was on generating high serum IgG titers we opted to use the intranasal prime and sub-cheek boost regimen, which generated the highest Env-specific IgG titers.

A robust CD8^+^ T cell response to HIV is one of the primary factors in controlling acute viremia and is almost always present in HEPS individuals; however, as HIV-1 continues to mutate, the CD8^+^ T cell response dwindles as it no longer recognizes many of the mutated HIV-1 epitopes [49–51]. CTL escape occurs during chronic HIV infection due to mutations and post-processing changes. Although often at the cost of reduced virulence, HIV, no longer kept in check by the CTL response thrives [52–54]. In our study, CD8^+^ T cells stimulated with Env and Gag peptide pools responded with a strong and consistent IL-2 cytokine production. Previous research has shown that CD8 IL-2 is attributable to continued expansion of memory CD8^+^ T cells [55]. This is corroborated by splenocytes stained for CD8, CD44, and CD62L, markers that distinguish naïve, effector, and memory T cells. The results showed an increase in CD8^+^ memory T cells in mice immunized with 0 {CALV(0)+VLP}, 7.5 {CALV(7.5)+VLP}, and 12.5 {CALV(12.5)+VLP}. The timing of our analysis is also important concerning the presence of IL-2, and the absence of IFN-γ. Both CD4^+^ and CD8^+^ memory T cells have been reported to primarily secrete IL-2, but not IFN-γ, cytokines that, when produced together, indicate non-lymphoid memory or general effector T cells [55,56]. Future studies would require analysis of both CTL function and an earlier time point to confirm that IFN-γ T effector cells were produced, but no longer detectable by week 3.

Finally, an effective vaccine must induce high IgG titers with specific binding to HIV, coupled with the ability of these titers to block HIV entry into CD4^+^ T cells through the CD4, CCR5, and/or CXCR4 receptors [57,58]. Our vaccine was capable of eliciting specific antibodies against HIV Env and Gag. With the addition of MPLA at 12.5 and 25 μg/dose, the concentration of IgG2c increased, while that of IgG1 remained the same, bringing the ratio of IgG1 to IgG2c close to 1. In line with these findings, MPLA has repeatedly been shown to induce a Th1-like or mixed Th1/Th2 like immune response when used as a vaccine adjuvant [18,59–61]. Recent research has indicated that IgG2a has greater binding activity to the high affinity Fcγ receptor and higher neutralizing activity *in vivo* than IgG1, when the two are genetically modified to have the same variable region [62,63]. Germinal centers, where affinity maturation and somatic hypermutation occur, are critical for the formation of neutralizing antibodies, which require additional mutations to recognize a broad spectrum of HIV clades [64,65]. The addition of 12.5 and 25 μg/dose of MPLA increased the number of germinal center B cells in the lymph nodes of our immunized mice. Therefore, the MPLA adjuvant served to both bolster a Th1-like response and increase germinal center formation.

It is important to note that only C57Bl/6 mice were used in this study. Compared to BALB/c mice, C57Bl/6 have two major immune differences that pertain to this study: 1) BALB/c mice have the IgG2a allele compared to C57Bl/6 mice which have the IgG2c allele; 2) BALB/c and C57Bl/6 mice often have opposite Th1/Th2 responses to various immunogens [66–69]. Although C57Bl/6 mice lack IgG2a, they have the phenotypically similar IgG2c (85% protein homology), which has been shown to be an allele IgG2a, with the two sharing a single locus in most mouse strains including C57Bl/6 [66]. Concerning Th1 and Th2, most research has compared the immune responses of C57Bl/6 mice with BALB/c mice after *Leishmania major* infection; with C57Bl/6 mice presenting a Th1-like response and BALB/c mice presenting a Th2-like response [68,70]. However, in the case of HIV envelope vaccines, both strains of mice have repeatedly been shown to exhibit very similar Th1-like immune responses [71–75].

In conclusion, we have introduced and characterized a novel route of vaccination, which is capable of inducing an immunoglobulin response that is more robust than those obtained using more traditional routes of intradermal and intranasal administration. Additionally, we have shown the importance of having an optimal MPLA concentration (likely ranging between 12.5 and 25 μg/dose) in VesiVax liposomes and of its role in inducing central memory T cells and germinal center B cells. These results are consistent with our vaccination studies on influenza virus [76,77] and herpes simplex virus 2 [78]. To confirm these findings, further studies with a higher vertebrate model are warranted.

## Supporting Information

S1 Fig Overview of sub-cheek administration. Vaccine was injected subcutaneously into each cheek at a final volume of 25 μl per cheek. (A) Anesthetized mouse before immunization. Sites of injection are marked with *. (B) Anesthetized mouse undergoing injection of vaccine. (C) Anesthetized mouse after vaccine administration.
(TIF)
