# Supplementary figures and images for: A Novel Prime and Boost Regimen of HIV Virus-Like Particles with TLR4 Adjuvant MPLA Induces Th1 Oriented Immune Responses against HIV
Source: PLoS One. 2015 Aug 27;10(8):e0136862. doi: 10.1371/journal.pone.0136862 (PMC4552547; doi:10.1371/journal.pone.0136862)

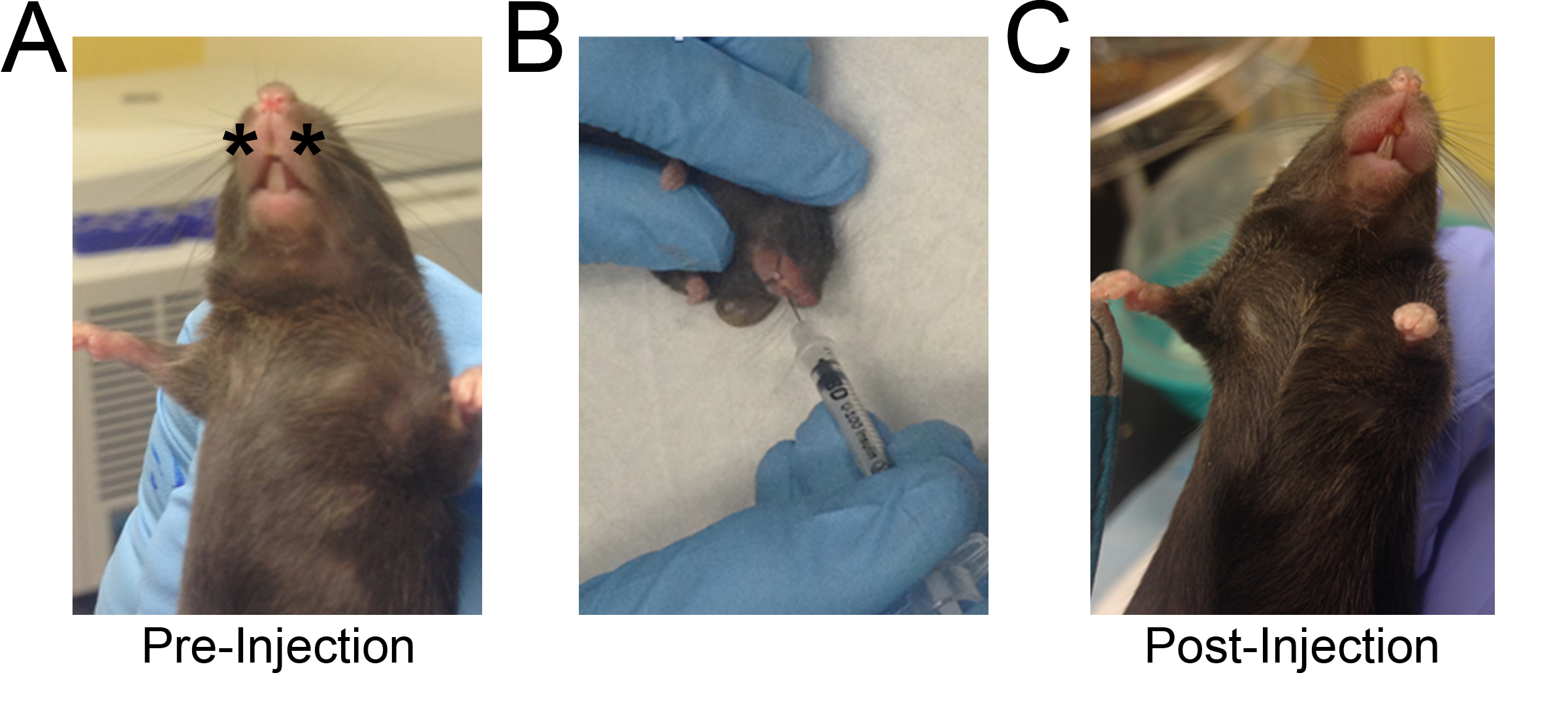

Supplement: S1 Fig — Vaccine was injected subcutaneously into each cheek at a final volume of 25 μl per cheek. (A) Anesthetized mouse before immunization. Sites of injection are marked with *. (B) Anesthetized mouse undergoing injection of vaccine. (C) Anesthetized mouse after vaccine administration. (TIF) [file pone.0136862.s001.tif]
